# Microstructure and Mechanical Properties of ZrB_2_–HfC Ceramics Influenced by HfC Addition

**DOI:** 10.3390/ma11102046

**Published:** 2018-10-20

**Authors:** Yi Jing, Hongbing Yuan, Zisheng Lian

**Affiliations:** 1 College of Mechanical Engineering, Taiyuan University of Technology, Taiyuan 030024, China; yuanhongbing@tyut.edu.cn (H.Y.); lianzisheng@tyut.edu.cn (Z.L.); 2 Shanxi Key Laboratory of Fully Mechanized Coal Mining Equipment, Taiyuan 030024, China

**Keywords:** ZrB_2_–HfC ceramics, hot pressing, sintering aid, microstructure, mechanical properties

## Abstract

ZrB_2_–HfC ceramics have been fabricated using the liquid phase sintering technique at a sintering temperature as low as 1750 °C through the addition of Ni. The effects of HfC addition on the microstructure and mechanical properties of ZrB_2_–based ceramics have been investigated. These ceramics were composed of ZrB_2_, HfC, Ni, and a small amount of possible (Zr, Hf)B_2_ solid solution. Small HfC grains were distributed among ZrB_2_ grain boundaries. These small grains could improve the density of ZrB_2_–based ceramics and play a pinning role. With HfC content increasing from 10 wt % to 30 wt %, more HfC grains were distributed among ZrB_2_ grain boundaries, leading to weaker interface bonding among HfC grains; the relative density and Vickers hardness increased, and flexural strength and fracture toughness decreased. The weak interface bonding for 20 and 30 wt % HfC contents was the main cause of the decrease in both flexural strength and fracture toughness.

## 1. Introduction

TheZrB_2_ ceramic is an ultrahigh-temperature ceramic with a high melting point (above 3300 °C), high electrical and thermal conductivity, high refractoriness, corrosion resistance, wear resistance, and ablation resistance [1,2,3]. Its physical and chemical properties make it a promising candidate for industrial applications in a harsh environment. However, the strength and fracture toughness of monolithic ZrB_2_ are very low, which limits its extensive applications. In order to expand its applications, ceramic additives such as SiC [4], ZrSi_2_ [5], (Ti, W)C [6], Co–WC [7], WSi_2_ [8], and ZrO_2_ fiber [9] have been employed into the ZrB_2_ matrix to overcome these defects.

The HfC ceramic also is an ultrahigh-temperature ceramic with a high melting point (about 3900 °C), high hardness, high electrical conductivity, and high elastic modulus and chemical stability [10,11,12]. HfC is a potential candidate material for aerospace applications, owing to its high melting point and low self-diffusion coefficient. These applications include scramjet components and rocket nozzles serviced at above 3000 °C [13,14,15]. Recently, it has emerged as a reinforcement phase to improve microstructure and mechanical properties for different ceramic matrix materials. HfC added into TiCN–based, TiB_2_–based, and ZrO_2_–based ceramics can form HfC particle dispersion that not only improves their microstructures, but also enhances their mechanical properties [16,17,18]. These reports provide a new approach to improve microstructure and mechanical properties of other ceramics through adding HfC. Over the years, numerous studies on the influence of Co–WC [7], WSi_2_ [8], ZrO_2_ fiber [9], MoSi_2_ [19], B_4_C [20], and SiC [21] additives on properties of ZrB_2_–based ceramic have been reported, whereas few studies on the effects of the HfC additive on properties of ZrB_2_–based ceramic have been undertaken.

ZrB_2_–based ceramics are commonly fabricated using the powder metallurgy technique. This technique mainly includes the solid phase sintering technique and liquid phase sintering technique. Compared to liquid phase sintering, solid phase sintering requires a higher sintering temperature. For solid phase sintering used in fabricating ZrB_2_–based ceramics, sintering temperature is usually about 2000 °C, such as 2200 °C for sintering ZrB_2_–TiB_2_ ceramics [22], 2150–2250 °C for sintering ZrB_2_–B_4_C ceramics [23], and 2000 °C for sintering ZrB_2_–SiC ceramics [24], to obtain complete densification. Simultaneously, such high sintering temperatures can promote ZrB_2_ grain growth that results in the reduction of flexural strength [25]. In order to obtain full densification of ZrB_2_–based ceramics and to lower the sintering temperatures, a hot pressing sintering process and metal sintering aids are usually employed in fabricating these ceramics. The addition of Fe to a ZrB_2_–SiC mixture makes it possible to decrease the sintering temperature from 2000 to 1600 °C [26,27]. Monteverde et al. pointed out that Ni can lower sintering temperature and promote densification of ZrB_2_–TiB_2_ and ZrB_2_–B_4_C ceramics during hot-pressed sintering [28]. Moreover, many studies show that the content of metal sintering aids is generally in the range of 4~10 wt % in the liquid phase sintering process of ceramics; too little metal content cannot promote the complete liquid phase sintering of ceramics; excessive metal content will lower the hardness of ceramics [29,30,31].

In this article, we apply the liquid phase sintering technique to prepare ZrB_2_–HfC ceramics at a lower sintering temperature through adding 8 wt % Ni. The effects of HfC addition on microstructure and mechanical properties at room temperature of ZrB_2_–based ceramics will be investigated.

## 2. Experimental Procedure

Commercially available ZrB_2_ powder (purity ≥ 99.8 wt %, C < 0.15 wt %, Fe < 0.09 wt %, Co < 0.011 wt %) with a median particle size of 1 μm from Northwest Institute for Non-ferrous Metal Research, Xi’an, China was employed. HfC powder (purity ≥ 99.9 wt %, O < 0.17 wt %, Fe < 0.08 wt %) with a median particle size of 0.8 μm from Shanghai Chaowei Nanomaterials Co., Ltd., Shanghai, China was used as secondary phase. Ni powder (purity ≥ 99.8 wt %, O < 0.06 wt %, C < 0.14 wt %, Fe < 0.06 wt %) with a median particle size of 1 μm from Qinhuangdao ENO High-Tech Material Development Co., Ltd., Qinhuangdao, China was used as a sintering aid. ZrB_2_–10 wt % HfC–8 wt % Ni (ZH10N), ZrB_2_–20 wt % HfC–8 wt % Ni (ZH20N), and ZrB_2_–30 wt % HfC–8 wt % Ni (ZH30N) were hot pressed at 1750 °C for 1 h under 30 MPa in a vacuum (3 × 10^−3^ Pa).

Before hot pressing, powders were mixed and milled for 72 h in a polyethylene jar with zirconia balls and alcohol as medium. Then, the mixed slurry was dried in vacuum and sieved by a 200-mesh sieve. After hot pressing, sintered samples were cut into test bars using the electrical discharge wire cutting method and the surfaces of the test bars were polished using diamond slurries. The dimension of the test bar was 3 mm × 4 mm × 50 mm. These test bars were cleaned by ultrasonication in absolute ethyl alcohol as a medium. After ultrasonic cleaning, these bars were dried for different tests. Ten test bars were tested for each experimental condition.

Flexural strength was measured at a span of 30 mm and across head speed of 0.5 mm/min using the three-point bending test method on an electron universal tester, according to Chinese National Standards GB/T 6569-2006/ISO 14704: 2000 [32]. Vickers hardness was measured on polished surfaces using a diamond pyramid indenter under a load of 196 N for 15 s by HV-120, based on Chinese National Standards GB/T 16534-2009 [33]. Fracture toughness (*K*_IC_) was measured via the direct indentation method [18]; the indentation was obtained through the Vickers hardness test. The density of each ceramic was measured using Archimedes’ method, with distilled water as a medium. Theoretical density was calculated according to the rule of mixtures, based on the following densities: 6.10, 12.7, and 8.90 g/cm^3^ for ZrB_2_, HfC, and Ni, respectively. Relative density was the ratio of the measured density to the theoretical density. An X-ray diffraction (XRD, EMPYREAN, PANalytical B.V., Almelo, The Netherlands) and energy dispersive spectrometer (EDS, ACT-350, Oxford Instruments, Oxford, UK) were used to analyze the compositions of the ceramics. A scanning electron microscope in back scattered electron mode (SEM and BSE, Supra-55, Carl Zeiss AG, Oberkochen, Germany) was used to observe the polished surface and fractured surface morphologies.

## 3. Results and Discussion

### 3.1. Effects of HfC Addition on Microstructure of ZrB_2_–HfC Ceramics

**Figure 1:**
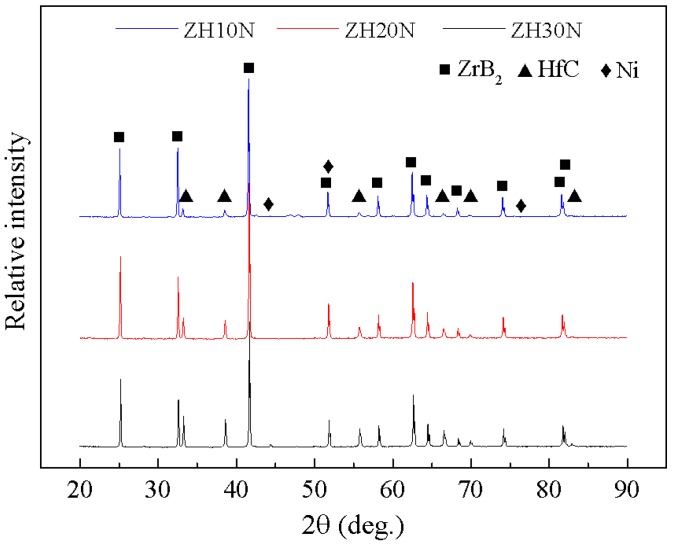
X-ray diffraction (XRD) patterns of ZrB_2_–HfC ceramics.

Figure 1 shows the X-ray diffraction patterns of ZrB_2_–HfC ceramics. The main crystalline phases were ZrB_2_, HfC, and Ni, which was consistent with the compositions of the raw powders. This indicated that no reaction occurred during the sintering process. It can be seen that as HfC content increased, the peak of HfC increased accordingly.

**Figure 2:**
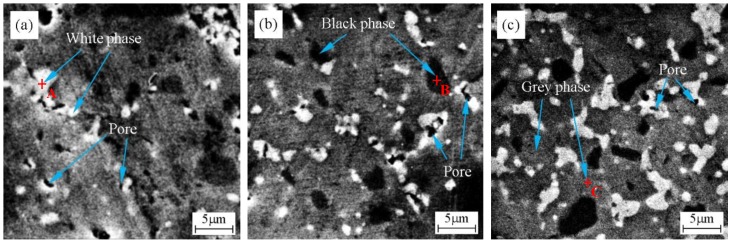
Scanning electron microscope in back scattered electron mode (SEM–BSE) micrographs of polished surfaces of ZrB_2_–HfC ceramics with different HfC content: (**a**) ZrB_2_–10 wt % HfC (ZH10N), (**b**) ZrB_2_–20 wt % HfC (ZH20N), and (**c**) ZrB_2_–30 wt % HfC (ZH30N).

**Figure 3:**
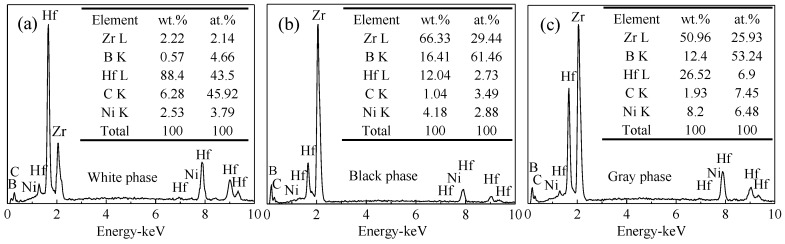
Energy dispersive spectrometer (EDS) measured at 30 kV of the phases in ZrB_2_–HfC ceramics: (**a**) EDS of point A for the white phase, (**b**) EDS of point B for the black phase, and (**c**) EDS of point C for the grey phase defined in Figure 2.

Figure 2 exhibits SEM–BSE micrographs of polished surfaces of ZrB_2_–HfC ceramics with different HfC content. Obviously, ZrB_2_–HfC ceramics had three phases: A white phase, a black phase, and a grey phase. In order to determine their compositions, they were separately subjected to EDS analysis. Figure 3 displays EDS results of points A, B, and C in Figure 2 for these three phases. In Figure 3a, the sum of mass fraction of the Hf and C elements was 94.68% higher than that of the other elements; moreover, the ratio of the Hf and C atomic fraction was 43.5:45.92 near to 1:1; according to XRD analysis results. Therefore, the white phase in Figure 2 was HfC. In Figure 3b, the black phase consisted of 66.33 wt % Zr and 16.41 wt % B, their total mass fraction was 82.74% higher than that of the others, and the ratio of their atomic fraction was 29.44:61.46 near to 1:2. Thus, based on XRD analysis results, the black phase in Figure 2 was ZrB_2_. In Figure 3c, the grey phase contained 50.96 wt % Zr, 12.4 wt % B, 26.52 wt % Hf, 1.93 wt % C, and 8.2 wt % Ni, indicating that the grey phase consisted of a mixture of all compounds. In fabricating ZrB_2_–SiC–HfB_2_ ceramics, Balak et al. pointed out that Hf and Zr diffused each other in a way that resulted in the formation of a (Zr, Hf)B_2_ solid solution [34]. Generally, when a new solid solution forms, twin peaks appear next to the standard XRD [35,36]. However, the peak position of ZrB_2_ and HfC in Figure 1 was in line with their respective peak position in the standard XRD card. According to the above analysis, the grey phase was probably a mixture of ZrB_2_ HfC, Ni, and a small amount of possible (Zr, Hf)B_2_ solid solution. Furthermore, there were some pores (marked in Figure 2) in ZrB_2_–HfC ceramics. These pores mainly derived from the sintering process and from grain pull-out during the procedure of grinding and polishing. Accordingly, the area of the white phase increased gradually with HfC content increasing in Figure 2.

**Figure 4:**
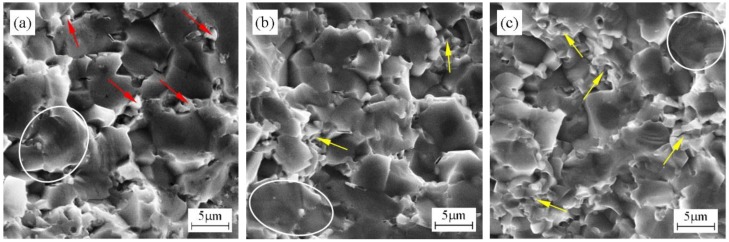
Fracture morphologies of the fracture surface of ZrB_2_–HfC ceramics with different HfC content: (**a**) ZrB_2_–10 wt % HfC (ZH10N), (**b**) ZrB_2_–20 wt % HfC (ZH20N), and (**c**) ZrB_2_–30 wt % HfC (ZH30N).

Figure 4 exhibits fracture morphologies of ZrB_2_–HfC ceramics with different HfC content. As can be seen, large ZrB_2_ and small HfC grains coexisted in these ceramics. The difference in the average ZrB_2_ grain size of these ceramics was very small. For each ceramic in Figure 4, the average grain size of ZrB_2_ was about 5 μm and of HfC was about 1 μm. These HfC grains were distributed among ZrB_2_ grain boundaries. In fabricating a ZrB_2_–SiC composite, Debnath et al. found that fine SiC particles distributed around ZrB_2_ grains could inhibit ZrB_2_ grain growth [27]. However, for ZrB_2_–HfC ceramics, these small HfC showed a small effect on ZrB_2_ grain growth. Generally, sintering temperature, additive content, and holding time are factors that determine the grain growth of the matrix phase. In the ZrB_2_–HfC–Ni system, the sintering temperature of 1750 °C perhaps had more significant effects on ZrB_2_ grain growth than HfC content and holding time; this resulted in just a small difference in the ZrB_2_ grain size. Moreover, compared to the size of raw powders, ZrB_2_ grain growth was obvious, from 1 to 5 um, while HfC grain growth was not obvious, from 0.8 to about 1 um, which also indicated that 1750 °C was more suitable for ZrB_2_ grain growth than for HfC. In HfC–SiC ceramics sintered at 2300 °C, Liu et al. pointed out that oxygen impurity can induce the growth of the HfC grain [37]. In this investigation, in order to reduce the introduced impurity, the mixed powders were milled in a polyethylene jar with zirconia balls and alcohol as mediums; then, the mixed slurry was dried in vacuum; after that, they were sintered in a vacuum (3 × 10^−3^ Pa). Therefore, in the fabrication processing, it was hard to introduce oxygen impurity to ZrB_2_–HfC–Ni ceramics, except the original oxygen impurity in raw powders. Compared to 1.7 wt % for the total content of oxygen impurity in HfC–SiC ceramics [37], 0.23 wt % for the total content of the oxygen impurity in ZrB_2_–HfC–Ni ceramics was considerably less; moreover, the sintering temperature of 1750 °C for fabricating ZrB_2_–HfC ceramics was lower than 2300 °C for fabricating HfC–SiC ceramics [37]; therefore, the unobvious HfC grain growth was ascribed to less oxygen impurity and lower sintering temperature in ZrB_2_–HfC–Ni ceramics. In addition, in Figure 4, there were a few coarse ZrB_2_ grains (as shown by circles) in each ceramic. These coarse grains were formed upon coalescence of small ZrB_2_ grains.

With HfC content increasing from 10 wt % to 30 wt %, more and more small HfC grains were located among ZrB_2_ grain boundaries. When HfC content was 10 wt %, many small HfC grains (as indicated by the red arrows in Figure 4a) were directly pinned into ZrB_2_ grains. These small HfC grains could play a pinning role to keep the material from fracturing during fracturing. This pinning effect of HfC grains on the ZrO_2_ grain boundaries was also discovered by Song et al. in ZrO_2_–HfC ceramics [18]. However, when the HfC content was 20 wt % and 30 wt %, some HfC grains (as indicated by the yellow arrows in Figure 4b,c) were distributed among the pinned HfC grains; these HfC hardly played the direct pinning effect on ZrB_2_; moreover, weak grain boundary strength may have been formed among these HfC grains due to the shortage of metal Ni to completely wet each of these HfC grains. Therefore, ZH10N would have stronger grain boundary strength than the other two ceramics.

### 3.2. Effects of HfC Content on Relative Density and Mechanical Properties of ZrB_2_–HfC Ceramics

**Figure 5:**
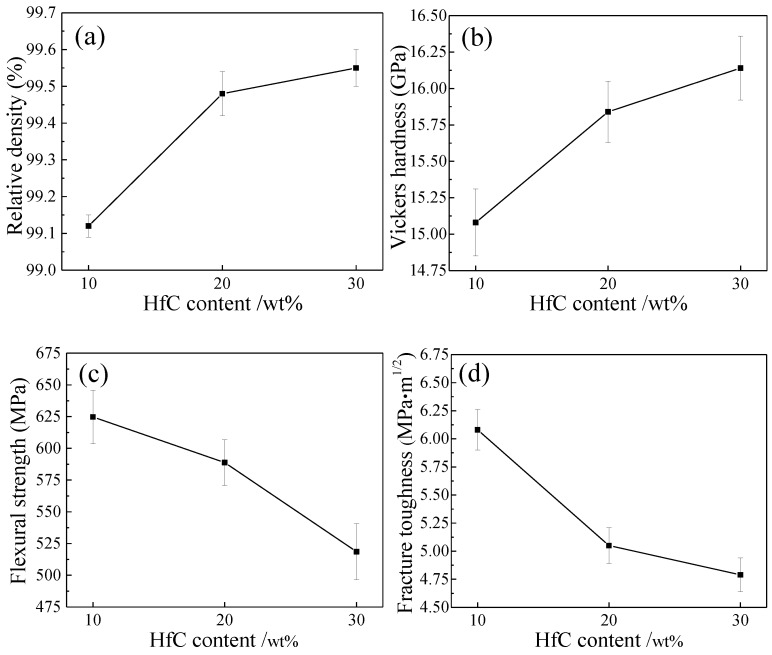
Relative density and mechanical properties of ZrB_2_–HfC ceramics with different HfC content: (**a**) Relative density, (**b**) Vickers hardness, (**c**) Flexural strength, and (**d**) Fracture toughness.

Figure 5 presents the relative density and mechanical properties of ZrB_2_–HfC ceramics with different HfC content. In Figure 5a, with increasing HfC content from 10 wt % to 30 wt %, the relative density gradually increased from (99.12 ± 0.03)% to (99.55 ± 0.05)%. During the liquid phase sintering, when HfC content was 10 wt %, the liquid metal Ni played a leading role in improving densification. The reason was that the liquid metal Ni would fill in gaps among grains. When HfC content was 20 wt % and 30 wt %, densification mainly depended on the increase of small HfC grains to fill in pores among grains. In precipitating, pores among grains would form because of big wetting angles of about 65° for ZrB_2_ and Ni, and about 50° for HfC and Ni [38]. With HfC content increasing, these small HfC grains would fill in these pores to improve the densification of ZrB_2_–HfC ceramics. Small particles or grains like SiC could also improve the densification of ZrB_2_–SiC ceramics, as reported by Debnath et al. [27]. The lowest relative density of the ZH10N ceramic ((99.12 ± 0.03)%) was higher than that of the monolithic ZrB_2_ ceramic (80.1%) sintered at 1750 °C, as reported by Sonber et al. [8].

Similarly, Vickers hardness slightly varied from about 15 to 16 GPa in Figure 5b. Numerous investigations [36,39,40] showed that, for ceramics, their Vickers hardness had a close relationship with their density: Generally, their Vickers hardness increased with their density increasing. ZrB_2_–HfC ceramics were no exception. In addition, the hardness of HfC (about 26 GPa) is higher than that of ZrB_2_ (about 22 GPa), which also promoted the improvement of its Vickers hardness with HfC content increasing. The Vickers hardness of the ZH30N ceramic (16.14 ± 0.22 GPa) was slightly lower than that of the ZrB_2_–MoSi_2_ ceramics (16.2 ± 0.5 GPa), as reported by Andrea et al. [41].

In Figure 5c, with HfC content increasing, their flexural strength gradually decreased from 624.72 ± 21 MPa to 518.58 ± 22 MPa. Generally, big grains resulted in the low flexural strength. However, the difference between the ZrB_2_ grain size of these three ceramics was very small, indicating that with HfC content increasing, there was another reason for the decrease in flexural strength. The reason was that with HfC content increasing from 10 wt % to 30 wt %, weak grain boundary strength formed among more HfC grains. Diffused weak grain boundary strength led to low flexural strength. The ZH10N ceramic had the highest flexural strength, owing to stronger grain boundary strength. The flexural strength of the ZH10N ceramic (624.72 ± 21 MPa) was higher than that of the ZrB_2_–MoSi_2_ ceramics (531 ± 46 MPa), as reported by Sciti et al. [42].

In Figure 5d, with HfC content increasing, fracture toughness gradually decreased from 6.08 ± 0.18 MPa·m^1/2^ to 4.79 ± 0.15 MPa·m^1/2^. In fabricating ZrB_2_–MoSi_2_ ceramics, through comparing the fracture toughness (2.3 ± 0.2 MPa·m^1/2^) of ZrB_2_–MoSi_2_ ceramics with that (2.8 MPa·m^1/2^) of monolithic ZrB_2_, Sciti et al. claimed that the addition of MoSi_2_ particles did not activate any toughening mechanisms, owing to the small difference in the coefficients of the thermal expansion between ZrB_2_ and MoSi_2_ (8.4 × 10^−6^/K) [42]. Moreover, in the ZrB_2_–SiC–TaSi_2_ system, Wang et al. assumed that with an increase in TaSi_2_ content, the weaker interface bonding between ZrB_2_ and TaSi_2_ caused by the different coefficients of thermal expansion between the ZrB_2_ (6.7 × 10^−6^/K) and TaSi_2_ (14 × 10^−6^/K) resulted in the improvement of fracture toughness [2]. However, compared to the toughening mechanisms of ZrB_2_–SiC–TaSi_2_ and ZrB_2_–MoSi_2_ ceramics, ZrB_2_–HfC had a different toughening mechanism. In this investigation, although the difference in coefficients of the thermal expansion between ZrB_2_ and HfC (6.6 × 10^−6^/K [18]) is very small, the lowest fracture toughness of ZrB_2_–HfC ceramics (4.79 ± 0.15 MPa·m^1/2^) was higher than that of monolithic ZrB_2_ (2.8 MPa·m^1/2^), which indicated that HfC addition played a toughening role. Additionally, the fracture toughness of ZH20N and ZH30N ceramics was lower than that of the ZH10N ceramic, which indicated that weak interface bonding cannot result in the improvement of fracture toughness. This weak interface easily caused the crack to propagate to produce a long crack that resulted in low fracture toughness. The fracture toughness of ZrB_2_–HfC ceramics mainly depended on the pinning effect of HfC on ZrB_2_ grains and the interface bonding strength among HfC grains.

## 4. Conclusions

ZrB_2_–HfC–Ni ceramics with 10–30 wt % HfC content were sintered at 1750 °C by hot pressing. The effects of HfC content on the microstructure and mechanical properties were investigated. The conclusions were as follow:

(1) ZrB_2_–HfC–Ni ceramics were mainly composed of ZrB_2_, HfC, and Ni. There were three phases: A white phase, a black phase, and a grey phase. The white phase was HfC, the black phase was ZrB_2_, and the grey phase was a mixture of ZrB_2_, HfC, and Ni with a small amount of possible (Zr, Hf)B_2_ solid solution.
(2) Small HfC grains were distributed among the ZrB_2_ grain boundaries. These small grains could improve the density of ZrB_2_–based ceramics and play the pinning role in these ceramics. ZrB_2_ grain growth influenced by HfC addition was not significant.
(3) With HfC content increasing from 10 wt % to 30 wt %, more HfC grains were distributed among ZrB_2_ grain boundaries, leading to weak interface bonding among HfC grains; the relative density and Vickers hardness increased and flexural strength and fracture toughness decreased. The weak interface bonding in ZH20N and ZH30N ceramics accounted for lowering the flexural strength and fracture toughness of these ceramics.
